# Microstructure of Sea Cucumber *Parastichopus tremulus* Peptide Hydrogels and Bioactivity in Caco-2 Cell Culture Model

**DOI:** 10.3390/gels11040280

**Published:** 2025-04-08

**Authors:** Miroslava Rossenova Atanassova, Jennifer Mildenberger, Marianne Doré Hansen, Tarmo Tamm

**Affiliations:** 1 Møreforsking AS, NMK, Borgundvegen 340, 6009 Ålesund, Norway; jennifer.mildenberger@moreforsking.no (J.M.); marianne.dore.hansen@moreforsking.no (M.D.H.); 2 Institute of Technology, University of Tartu, Nooruse 1, 50411 Tartu, Estonia; tarmo.tamm@ut.ee

**Keywords:** sea cucumbers, *de novo* peptides, hydrogel formation, microstructure, electron microscopy, antioxidant activity, angiotensin-I converting enzyme inhibitory activity, Caco-2, protection against oxidation in cell culture

## Abstract

Wider availability of marine proteins for the development of food and biomedical applications has a high importance. Sea cucumber body wall proteins have specific functional properties that could be very promising for such product development. However, protein extraction from whole animals is costly and complex, whereas peptide hydrogel production using biotechnological methods can be considered an economically viable approach. Body-wall derived peptides from sea cucumber *Parastichopus tremulus* have been suggested as a nontraditional source of potentially edible hydrocolloids. In the current work, four peptides were produced through custom synthesis. Scanning electron microscopy (SEM) of the combined mix of the four peptides (1:1 ratio; 15 mM concentration) in a calcium ion-containing buffer confirmed untargeted self-assembly with long, thick fibrillar formations at a microscale (measured mean cross-section 2.78 µm and length sizes of 26.95 µm). The antioxidant activity of the peptides separately, and in combination (1:1 molar ratio), was studied *in vitro* through ORAC (values in the range from 279 to 543 µmol TE/g peptide), ABTS (from 80.4 to 1215 µmol TE/g peptide), and DPPH (from 5.2 to 19.9 µmol TE/g) assays, and confirmed for protection against oxidation in a Caco-2 cell culture model. Angiotensin-I converting enzyme inhibitory activity was also confirmed for two of the four peptides, with the highest IC 50 of 7.11 ± 0.84 mg/mL.

## 1. Introduction

The extracellular matrix (ECM) of echinoderms has been a central focus of research and extensively studied over the past 50 years, due to its unique mutable collagenous tissue (MCT). In the holothurian ECM, collagen fibers, composed of fibrillin-like proteins, proteoglycans, fucosylated chondroitin sulfate, tensilin, stiparin, novel stiffening factor (NSF), matrikines, and various other small molecules have been reported [1,2]. Collagens and proteoglycans constitute the primary structural components, yielding together about 70% of the total body wall composition [2]. The full composition of the body wall ECM for sea cucumber *Apostichopus japonicus*, for which an annotated proteome already exists, has been reported [3]. The most advanced microscopic analyses of the sea cucumber ECM so far have been performed on *Holothuria scabra* [4], *Apostichopus japonicus* [5], *Holothuria leucospilota*, and *Stichopus chloronotus* [6]. MCT has inspired the design of different biomimetic (mostly artificial, but biocompatible) materials and medical devices and remains a key area of research, particularly in studying the molecular mechanics underlying the observed tensile changes in the holothurian body wall [1]. Sea cucumber collagens (from species *Stichopus hermanii*, *Holothuria tubulosa*, and *A. japonicus*) have been demonstrated to serve as essential components in biomimetic materials for human tissue regeneration and wound healing [7]. Sea cucumber glycosaminoglycans (GAGs) and polysaccharides, as separate biopolymer groups, have been well characterized regarding their structural organization and bioactivities [8].

Decellularized extracellular matrices (dECMs) are another group of highly attractive biomaterials, used for bioprinting and the creation of complex tissue constructs, supporting the growth of a wide range of cell cultures [9]. Extracellular matrix-based hydrogels and microspheres have even greater potential for novel biocomposite development, with a wide spectrum of final applications [10]. No sea cucumber dECM biocomposites have been reported so far. On the other hand, small sea cucumber-derived peptides (e.g., with ECM-mimicking properties) offer a broad functional property range [11], have a well-demonstrated ability to self-assemble into larger structures (hydrogels), and are currently being used for the design and construction of specific biomaterials—nanostructures, particle carriers, and tissue culture scaffolds [12,13,14]. Special research interest has been placed recently on matrikines (MKs)—bioactive molecules with a peptide nature, obtained only after the proteolytic/autolytic degradation of the body wall proteins of sea cucumbers [2]. Most anti-inflammatory MKs have low molecular weights (≤1000 Da) and are rich in glycine, glutamate, and aspartic acid. A recent study in the red sea cucumber *Parastichopus tremulus* [15] has reported an antioxidant capacity of peptides obtained after different phases of simulated human digestion *in vitro*, but without using a cell culture or tissue model for the evaluation of the absorption of nutrients or bioactives. In a previous study on the bioactive peptide composition of enzymatically digested sea cucumber *P. tremulus* body wall fractions, we have reported the *in silico* predicted capacity of some of these peptides to self-assemble into fibrous structures [16].

Bioinformatics and computational (sequence-based molecular docking) methods like AlphaFold 3 [17] have been used with increasing success in the recent years, to model and predict the functionalities of self-assembled structures, for ligand binding and the targeted biodiscovery of novel functional molecules with enhanced bioactivity. With reference to sea cucumbers, a recent publication has focused on the *in silico* analysis of bioactive peptides from underutilized *Cucumaria frondosa* mouth parts and internal organs [18]. The PEP-FOLD 3 de novo peptide structure prediction engine [19], based on a new Hidden Markov Model sub-optimal conformation sampling approach, has shown a high capacity to generate 3D structural models for peptides from 5 to 50 amino acids. This capacity of PEP-FOLD3 was already used by Wargasetia and colleagues [20] for molecular docking and 3D structural estimation of sea cucumber *C. frondosa* peptides’ fitness to block three different proteins important for breast cancer growth. However, experimental proof from a broad range of methodologies is still required to confirm the predictions of machine learning for unstudied organisms, and to enable novel food or biomedical product development.

Despite all the promising findings, some of which already support commercial biomaterial and food production, there are many marine organisms that have not been well studied. The sea cucumber species *P. tremulus* is among them. These insufficiently studied organisms might still prove to be novel sources for material and product development in a wider range of functional and health applications. The current study focuses on experimentally confirming the predicted *in silico* gelation properties (fibril formation) and bioactivities of four de novo peptide sequences obtained from the sea cucumber *P. tremulus* body wall [16]. We have, thus, first obtained the peptides in their pure form through a synthetic approach; then, secondly, determined the limiting concentrations of the peptides for gel structure observation using microscopy; and, thirdly, tested the individual and combined peptide bioactivities *in vitro*, and in a Caco-2 cell culture model. The bioactivity, self-assembling capacity, corresponding microscopic structures, and hydrogel formation by the selected sea cucumber peptides have been studied here for the first time in view of their possible application as components of more complex hydrogel formulations and ECM structures. No previous publications have explored hydrogel development with the selected peptide sequences, as part of dECM biocomposite development.

## 2. Results and Discussion

The four de novo peptide sequences chosen for this work (Table 1) have already been aligned with existing evolutionarily related sea cucumber (*Apostichopus japonicus*) and sea urchin sequences (*Strongylocentrotus purpuratus*). Partial similarities have been found with sections from ECM fibrillar proteins [16]. We started with the hypothesis that if the synthetic peptides, prepared on the basis of these sequences, could be efficiently combined in a dissolution, they could form fibrillar-type, supramolecular structures, above a critical concentration and in defined conditions. Additionally, if their expected bioactivity was confirmed, it could be preserved to a certain extent in the hydrogel system and impart an increased cell survival support capacity to the corresponding fibrillar structures, if used for scaffolding.

### 2.1. In Silico Modelling of the Expected 3D Structure of the Peptide Gel in Presence of Chelators

**Figure 1:**
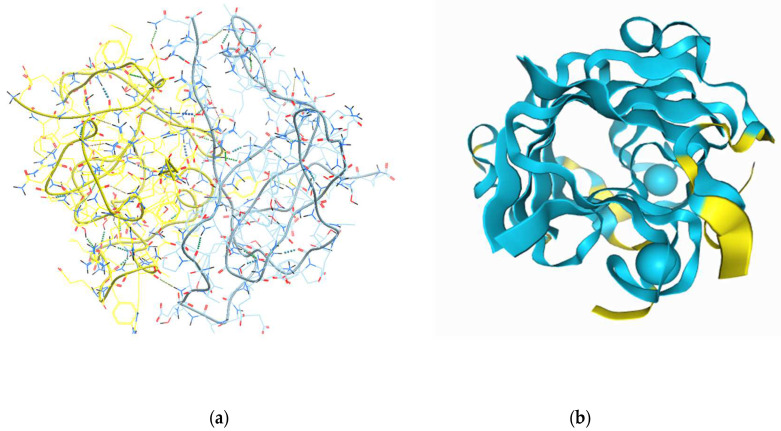
Model of the most probable 3D structural unit to be formed of the interactions of the four peptide sequences from *P. tremulus*, in two different graphic modes: (**a**) in MolSoft Browser v3.9, the display of the backbone of the dimeric molecule with hydrogen bonds and AA residues; each dimer is colored in a different color; (**b**) in the AlphaFold 3 browser, with the inclusion of the predicted folding for the dimeric complex of the four peptide sequences and the possible interaction sites with the Ca^2+^ ions (presented as blue balls, interacting with the sequence of P4 in each monomer of the presented dimeric supramolecular structure). The different colors of the molecular parts signify the different structural probabilities (blue—structural confidentiality between 90 and 70%, yellow—between 70 and 50%).

*In silico* modelling was carried out in the conditions of a neutral pH with two modelling software applications. The results for the most viable structural interaction model, predicted using the Galaxy Web server (https://galaxyweb.seoklab.org), for all the combinations of the selected sea cucumber peptide sequences, are summarized in Figure 1a. Homomer (protein homo-oligomer structure prediction from a monomer sequence or structure) and heteromer (protein hetero-dimer structure prediction from sequences or structures of subunits) modelling options were tested for all the sequence combinations. A fitting template was identified for seven out of all the possible homomer peptide combinations and for four out of all the heteromeric peptide combinations using the template-based docking (TBD) algorithm. The templates with which homologies were detected belong to sections of the active sites of known enzymes, with a special interest in the alignment of combined four-peptide sequences to sections of a cyanocobalamin complex, which supports the previously observed metal ion complexation capacity. The highest TBD score was yielded for the homodimeric combination of all the peptides (987.30), followed by the heterodimeric structure formed by the 1:1:1:1 ratio combination of all four peptide sequences (848.84). The Galaxy Web software did not allow for the inclusion of metal chelating ions in the modelling process at the time of accession for our study.

The prediction for the random organization of the peptide complexes and the influence of the Ca^2+^ ions on their 3D structure could be modeled using the AlphaFold 3 server (https://alphafoldserver.com), which was recently validated for Open Science use. The result of this modelling effort is shown in Figure 1b. The model presented in the figure is of a dimeric structure (two copies from each peptide sequence and two calcium ions) and its interface predicted template modeling score (ipTM) is 0.57–0.59. This score is derived from the template alignment measure for the accuracy of the interface predictions in the entire structure [17]. The ipTM values for the complex modelling we applied were below the high confidence threshold (0.8) since the structural information available for the proteins, from which the selected peptides were a part, was generally very limited. Also, the obtained lower modelling score could be due to the current lack of reliability in the predictions for the flexible regions of proteins in Alpha Fold 3 [21], and to the relatively small sizes of the peptides studied by us.

*In silico* modelling of secondary and tertiary protein structures based on experimentally confirmed molecular templates, e.g., the ones obtained from crystallography, X-ray diffraction, electron microscopy, small-angle scattering, solid-state nuclear magnetic resonance, or 3D Fourier-transform spectroscopy [22,23], provides the highest accuracy. However, such detailed structural information is usually obtained after complex analytical studies, which are not available for most proteins from generally understudied organisms. As an example, in the Worldwide Protein Data Bank (wwPDB) and the Research Collaboratory for Structural Bioinformatics Protein Data Bank (RCSB PDB) archive, there are currently deposited only 16 experimental structures of proteins derived from holothurians, 6 for proteins from sea urchin *Paracentrotus lividus*, and 13 for those from *Strongylocentrotus purpuratus*, compared with a really high number of proteins derived from *Homo sapiens* or bacteria (statistics accessed in November 2023). Interestingly, no homologies were automatically detected by the used-by-us web-based software tools with the known structures in related marine echinoderm species. Since there is a lack of publicly available biological information at the molecular level for sea cucumber *P. tremulus*, we selected the ones derived from the highest TBD score models as the most probable interactions, i.e., the homo- and heteromeric complexes to be formed by all the peptides in 1:1:1:1 ratio combination. These interaction models were further used as the basis for the preparation of gels and their structural analysis using SEM.

### 2.2. Experimental Solubility and Minimum Gelation Conditions

According to the manufacturer of the four custom peptides, the solubility of peptides with more than 25% charged residues is expected to be generally good at a neutral pH in aqueous systems (rule applying mainly to custom peptide 3, and partially to peptide 2), while the peptides with sequences containing high or very high hydrophobic residue numbers (over 50 or 75% of the total number), would have low or no solubility in aqueous buffers (peptides 1 and 4). For these highly hydrophobic peptides, the addition of dimethyl sulfoxide (DMSO), acetonitrile, or dimethylformamide (DMF) was advised (in a ratio of up to 50% of the solution) even if these agents could damage the amino acid residues that are sensitive to oxidation (C, M, W). These solubility conditions were taking into consideration the degree of purity of the different peptides after the HPLC analysis by the producer. Therefore, we used these as the basis for selection of the initial conditions in the gelation trials (Table 2). After making charge calculations based on the sequences of the peptides (considering also the N-terminal acetylation), the net amino acid residues at pH 7 for most of the peptides (1, 3, and 4) corresponded to acidic charges, while peptide 2 was expected to behave with a net basic charge in such conditions. The solubility of the peptides was tested in the two selected neutral buffer systems (with and without Ca^2+^ ions), which are normally abundant in sea cucumbers and known previously to cause the chelation of sea cucumber peptides [16,24].

The individual peptide solubility of peptide 2 (sequence Ac—RAGQPITAFLVRD) was low in both buffers. DMSO had to be added in different concentrations to the peptide 1, 2, and 4 solutions, when these were resuspended in the Ca^2+^-containing buffer (2.5% *v*/*v*, 5% *v*/*v*, and 2.5% *v*/*v*, correspondingly). After 10 min at room temperature, all the separate solutions of peptides 1, 2, and 4 (at a 1 mM final concentration) in the Ca^2+^-containing buffer system formed gels with different optical characteristics: nontransparent, white cream-like gel (peptide 1), nontransparent (whitish yellow) solid gel (peptide 2), and transparent solid gel (peptide 4). Peptide 3 was fully soluble and did not form a gel in these conditions, but only after increasing the peptide concentration to 15 mM. A further targeted optimization of the gelation conditions (e.g., temperature rise, pH shift or higher Ca^2+^ ion concentrations) would be needed for separate peptide dissolutions.

In the sodium phosphate buffer (NaPB), at pH 7.4, peptides 1, 3, and 4 dissolved after gentle agitation without any need for DMSO addition, with experimentally determined pH 6.5–7 in the concentration range of 1–15 mM. Gelation for these three peptides would occur in the tested experimental conditions only at concentrations above 15 mM. P2 was insoluble in this system until initial resuspension in DMSO (for 7.5% *v*/*v* final content), followed by the addition of the aqueous buffer. However, in this case, depending on the concentration of peptide 2, gelation would occur in DMSO during dissolution. The same was applied to peptide 4, if initial resuspension in DMSO was attempted, prior to the addition of the phosphate buffer.

Equal ratios of the peptides were tested in the all-peptide mix, based on the results of the *in silico* modeling (Section 2.1) and the model presented in Figure 1, where the structure appears to be stabilized by the complexation of two molecules from each peptide (dimeric structures). The suspension of the mixed peptides was much easier to solubilize in both buffers than the individual peptides, not requiring DMSO for gelation in the Ca^2+^-containing buffer, which occurred spontaneously after 1 min of agitation.

Thus, the minimal gelation conditions for the four-peptide mix were defined as a 15 mM peptide concentration and a 1:1:1:1 ratio, in the presence of Ca^2+^ ions. These conditions yielded a solid transparent gel (Supplementary Figure S2), after vortexing at room temperature. The resulting gel was freeze-dried and submitted to scanning electron microscopy (SEM) and other structural analyses. By combining the studied peptides with some of the widely used polysaccharide biopolymers, the gelation conditions can be further optimized, to mimic the known ECM organization in the sea cucumber body wall [1]. Similar gelation conditions (pH and Ca^2+^ concentration dependent) have been also reported for supramolecular polymer (SP) hydrogels based on sea cucumber dermis [22] with differential mechanical properties (rigid and stable at pH 3/high Ca^2+^ concentration or forming soft networks at alkaline pHs in the lack of Ca^2+^ ions), which had an autolytic capacity at a physiological pH.

### 2.3. Structural Characterization

#### 2.3.1. SEM Analysis

**Figure 2:**
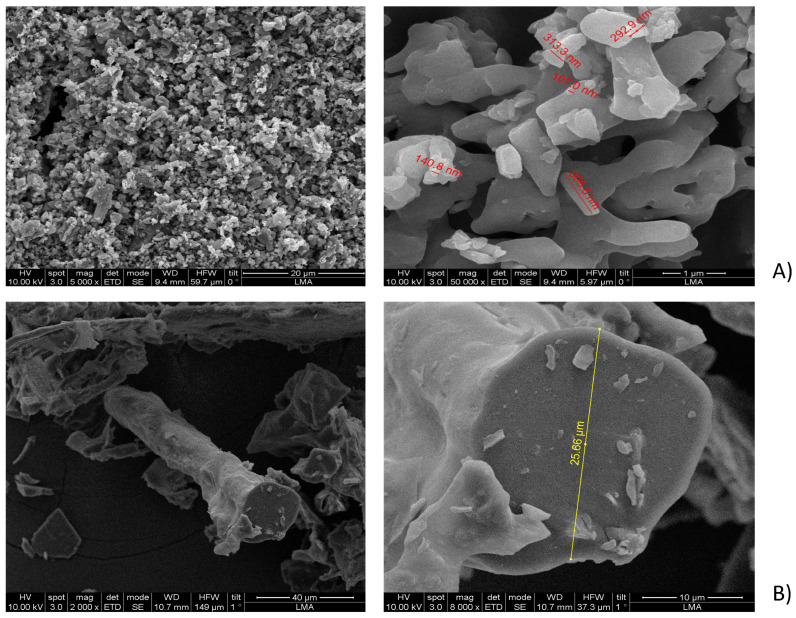

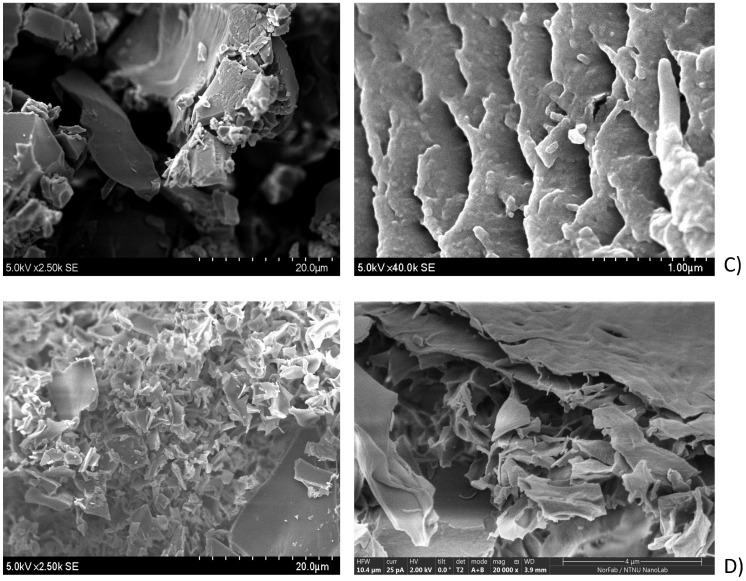

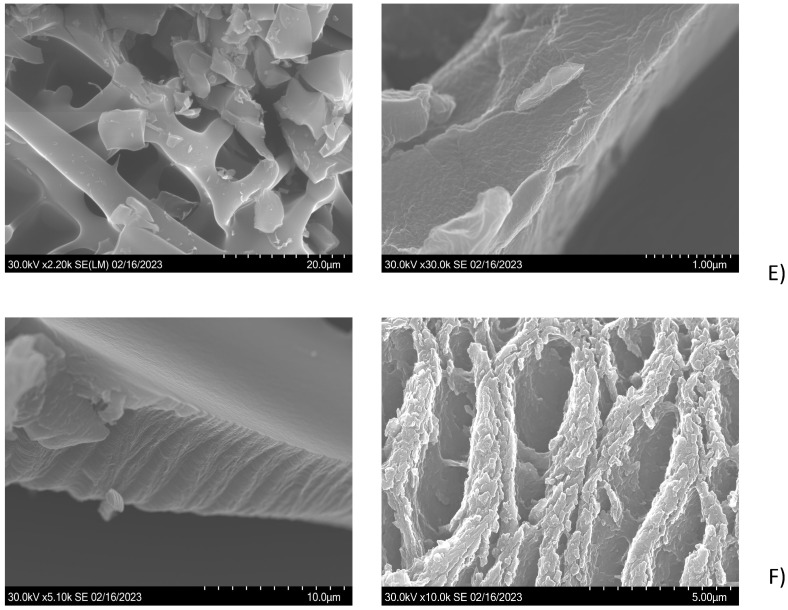
SEM micrographs showing the observed structure of the freeze-dried sea cucumber *P. tremulus* peptide hydrogels, at one higher and one lower magnification in each case (higher magnification image always on the right hand side of the figure subsections): (**A**) Cryo—SEM of the initial mixed fraction of sea cucumber peptides, isolated using size exclusion chromatography on FPLC from the sea cucumber enzymatic hydrolysate (at 20 µm and 1 µm); (**B**) Cryo—SEM of the mixed fraction of four custom synthesized sea cucumber peptides in ratios of 1:1:1:1 (at 40 µm and 10 µm); (**C**) Peptide 1 after gold coating (at 20 µm and 1 µm); (**D**) Peptide 2 after gold coating (at 20 µm and 4 µm); (**E**) Peptide 3 after gold coating (at 20 µm and 1 µm); (**F**) Peptide 4 after gold coating (at 10 µm and 5 µm). Additional images are included in the Supplementary Materials, Figure S3.

The SEM analysis of the initial (purified by FPLC, bioactive) fraction, isolated by us from sea cucumber hydrolysate, showed particles with variable sizes, ranging between 240 nm and 4.31 μm (Figure 2A; Supplementary Table S1), and nonhomologous morphology (amorphous structure). In contrast, the mixture of the four custom synthesized peptides after gelation showed a definite fibrous structure (Figure 2B), which could correspond to the linear complexation of the subunits, similar to the model prediction in Figure 1b. The gel with the four-peptide mixture contained long fibrillar formations with a cross-section of approximately 2.78 µm and a length of 26.95 µm. The smaller particle sizes and forms, observed in the case of the initial complex fraction, isolated from the hydrolyzed sea cucumber biomass using FPLC, could be explained with the lack of fibril formation and the presence of many different peptides that influenced the supramolecular organization during lyophilization.

The particle morphology of each separate peptide gel after lyophilization was quite different (Figure 2C–F), although clear laminar organization was appreciated in the microstructure of all the peptide gels. The sizes of the particles, formed at the same gelation conditions by the different peptides, were ranging between 4.3 and 17.7 µm in length, while their cross-section varied between 2.0 and 4.3 µm. Additionally, peptides 1, 2, and 4 showed fiber arrangements into ECM-like structures (Figure 2C,F; [25]). In peptide 3, the arrangements of the particles also mimicked fibrillar structures, while peptide 2 showed a non-homogeneous layered organization, similar to epidermal connective tissue in other sea cucumbers (e.g., *H. scabra* [26]; *A. japonicus* [27]). The size measurements both for length and cross-section (Table S1) showed statistically significant differences among all the samples (single-factor ANOVA for the cross-section with *p* ≤ 0.008 and for length with *p* ≤ 8.7075 × 10^−13^. The comparison between the particle sizes in the initial FPLC fraction and in all the samples prepared with the synthesized peptides showed significant differences (Table S1b), while there were no significant differences in the particle cross-section (diameter) between the mix of the four peptides and the individual peptide preparations. The lengths of the fiber structures in the samples were significantly different, except in the case of peptides 2, 3, and 4, which showed clustered length size values (Table S1b). The size of the collagen fibrils in sea cucumbers will generally depend on the state of the connective tissue (stiffened/softened) and on the type of connective tissue, the structure of which varies depending on the originating organs and species [3]. The collagen fibers in the various sea cucumber body wall dermal layers are usually arranged in laminar superstructures, with an alternating orientation of the fibrils, so these are seen sometimes as round cross-sections and sometimes as longitudinal views. Such laminar structures have been reported for *Screrodactyla briareus*, *Eupentacta quinquesemita*, or *Holothuria forskali* [28]. Our measurements for the cross-section (diameter) of the fibers, formed in the gel of the four peptides together, show higher values in comparison with the previously reported ones for both stiffened and softened states. For example, in dense connective tissue (DCT) layers in sea cucumber *E. quinquesemita*, the collagen fibrils with diameters 20–160 nm, organized in small bundles or as single fibrils have been reported [29]. The collagen fibrils in the loose connective tissue (LCT) of the same species have a 40–60 nm diameter. In muscle tendon, the collagen fibrils have diameter of about 30–40 nm and the microfibrils of 10 to 15 nm. The mean length measured by us in the combined peptide gel, however, is comparable with the transmission electron microscopy (TEM)-measured collagen fibril length in the body wall of *E. quintesemita* [29].

**Figure 3:**
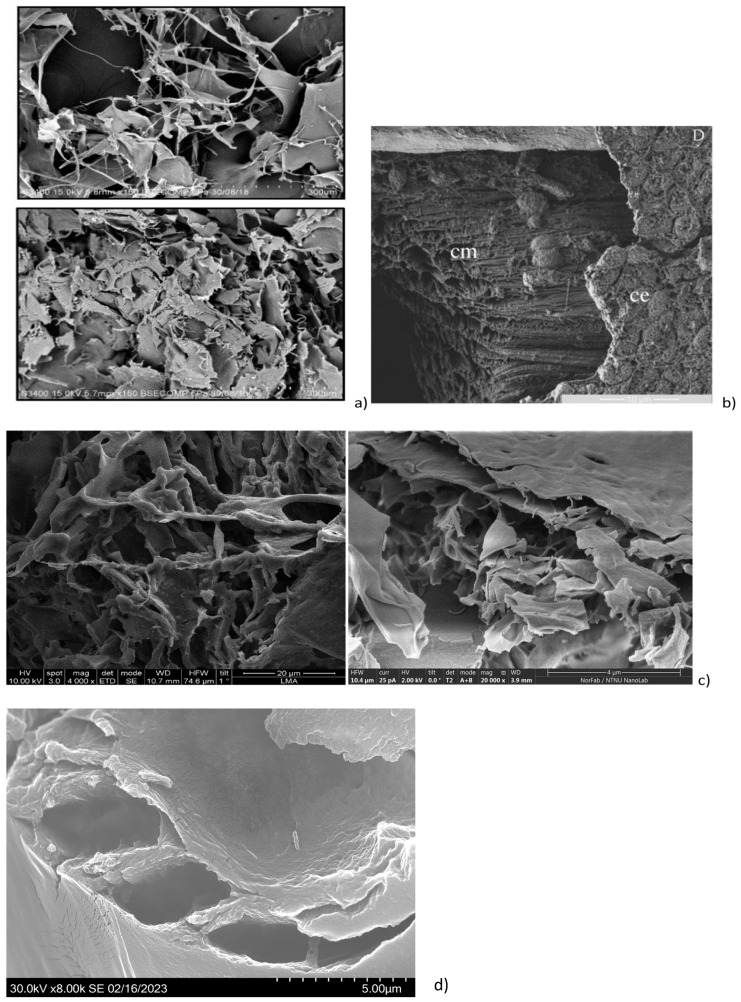
Comparison of the observed lyophilized peptide microstructures from *P. tremulus* with previously reported SEM micrographs in sea cucumbers *H. scabra* and sea urchin *P. lividus*: (**a**) SEM images of the dialyzed and ultrafiltered collagen isolates from *H. scabra* (reprint from [4]; Copyright 2021 MDPI Journals); (**b**) SEM micrograph of a milled sea urchin *P. lividus* ligament with collagen bundles (cm) covered by myoepithelium (reprint from [33]); (**c**) SEM images from the mixed lyophilized gel of the four sea cucumbers *P. tremulus*—derived body wall peptides; (**d**) SEM image from the peptide 4 lyophilized gel. The image from [33] is reproduced under the terms and conditions of the Creative Commons Attribution (CC BY) license (http://creativecommons.org/licenses/by/4.0/). Higher magnification images are on the right side of the figure sections b and c.

**Figure 4:**
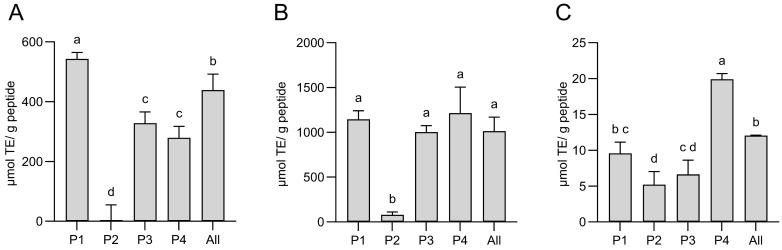
Antioxidative capacity of the synthesized sea cucumber peptides from *P. tremulus*, as determined by biochemical assays *in vitro*. (**A**) by ORAC; (**B**) by ABTS; (**C**) by DPPH assay. The results are expressed as mean Trolox equivalents (TEs) ± standard deviation of three replicates and the differences were tested using ANOVA, followed by the Holm–Šídák method. The same superscript letter means that the difference between samples is not significant, while bars with different superscript letters are significantly different from each other (*p* < 0.05).

The morphology of the initial FPLC fraction with a multiple peptide composition has clear similarities with that of the sea cucumber powder from *H. scabra*, obtained after thermal treatment (10 min at 80 °C) and air-drying [30]. The microstructures observed by us for the mix of the four synthesized peptides, as well as for peptides 2, 3, and 4 were also similar to the ones obtained using SEM for the collagens from the body wall of sea cucumber *H. scabra* (Figure 3a, [4]), as well as for the soluble collagen fraction from the *A. japonicus* body wall (treated with an endogenous complex protease) (Figure 3b, [31]). Overall similarities can also be observed to the microstructure of the collagen matrix fibrils (collagen bundles under the coelomic epithelium) in sea urchin *P. lividus* (Figure 3c, [32,33,34]). Finally, from the microstructural study of the gel, formed by the mixture of the four synthesized peptides (at 15 mM and in 1:1 ratio), we see that self-assembly occurs spontaneously and results in tubules, even if the process is apparently random. The possible covalent crosslinking effects of DMSO or methanol (besides the non-covalent interactions introduced by Ca^2+^) should not be disregarded but could have contributed to the formed structures only in the case of the individual peptide gels. For the resuspension of the peptides in the four-peptide mix, no addition of DMSO or other agents was necessary. Further detailed structural studies are needed to finetune the conditions for targeted assembly, with controlled structure–function relationships, for example, for 3D printable bioink development.

#### 2.3.2. UV Spectroscopic Analysis

UV spectroscopy is widely used for the analysis of peptide structures due to the ability of the side chain groups of specific amino acids to absorb UV light in a certain wavelength range. The knowledge of the peptide composition assists the estimation of the expected spectra, although the interactions among the peptides in the solution would also influence the peak of the measured spectra. The luminescent amino acids in proteins are mainly phenylalanine, tyrosine, and tryptophan, with the fluorescence peaks at around 282 nm, 303 nm, and 348 nm, respectively [35]. All the sea cucumber peptides (at concentrations of 5–25 mg/mL) presented absorption peaks within two wavelength ranges: 220–240 nm and 280–300 nm (Figure S4), which aligned with the corresponding fluorescent amino acid composition per peptide. The mix of the four peptides at a concentration below the minimum gelation concentration (12.5 mg/mL) showed similarity with the single peptides in the wavelength range of 233–365 nm. However, the maximum absorbance intensity increased slightly in the peptide mixture (recorded additional peak at 376 nm), which proved that the secondary structure of the peptides had changed due to new bond formation and possible aggregation interactions. The region of the UV spectrum of 200–230 nm is known to be related to peptide bonds, while the higher wavelengths are related more to the aromatic regions of the amino acids and intermolecular hydrogen bond complexation (310 nm and above) [35].

### 2.4. Experimentally Determined Bioactivities

The synthetic peptides are expected to have a significant physiological role in any of the supramolecular constructs for enhanced cell viability or food hydrocolloid systems, and thus, their bioactivity required further screening. Based on the predicted bioactivity types for the peptide sequences and the previous antioxidant activity results in the complex FPLC fraction from *P. tremulus* hydrolysate, we have screened for antioxidant and angiotensin-I converting enzyme inhibitory activities *in vitro*.

#### 2.4.1. Measurement of the Antioxidant Capacity

In the ORAC assay, all peptides except peptide 2 showed antioxidant activity. The highest activity was shown by P1 (543 µmol TE/g protein) and was somewhat lower for P3 and P4 (328.5 and 279 µmol TE/g protein, respectively) (Figure 4A). P2, which was dissolved in DMSO due to insolubility in the aqueous ORAC buffer, had no detectable activity at the dilutions required to overcome the interference of DMSO in the assay. The measured activity for P1, P3, and P4 at this concentration is lower than previously reported for the initial *P. tremulus* hydrolysate (up to 791 μmol TE/μg protein) and derived FLPC fractions (about 400 μmol TE/mg protein) [16]. Peptide fractions from *A. japonicus* selected for their high antioxidant activity have been reported with ORAC values of 17.48 μmol TE/mg peptides [36]. It must be noted that the synthesized peptides were primarily chosen for our study due to their predicted high self-assembling capacity, rather than for their bioactivities, and their antioxidant capacity was tested to experimentally confirm the expected results from the *in silico* prediction bioactivities [16]. The antioxidative capacity assessed using the ABTS assay was similar for P1, P3, and P4 with 1003–1215 µmol TE/g protein. The activity of P2 was significantly lower, with 80.4 µmol TE/g, which could again be due to solubility differences (Figure 4B). The overall activity was lowest in the DPPH assay, ranging from 5.2 to 19.9 µmol TE/g for all the peptides, with P4 having significantly higher activity. There was no synergistic effect detected in the combination of all the peptides in any of the tested antioxidant assays. The observed lack of synergy among the peptides in the mixture could be partially explained by the observed solubility differences among the peptides in the buffered systems, used for the *in vitro* antioxidant activity evaluation. On the other hand, the expected interactions among the peptides in the solutions (even if not at the minimal gelation concentration) could also be responsible for the bioactivity reduction. The structural reassembly and blocking of bioactivity in the responsible molecular sections during protein–protein interactions at physiological conditions are well documented [37].

#### 2.4.2. Measurement of the Angiotensin-I Converting Enzyme Inhibitory Capacity

**Figure 5:**
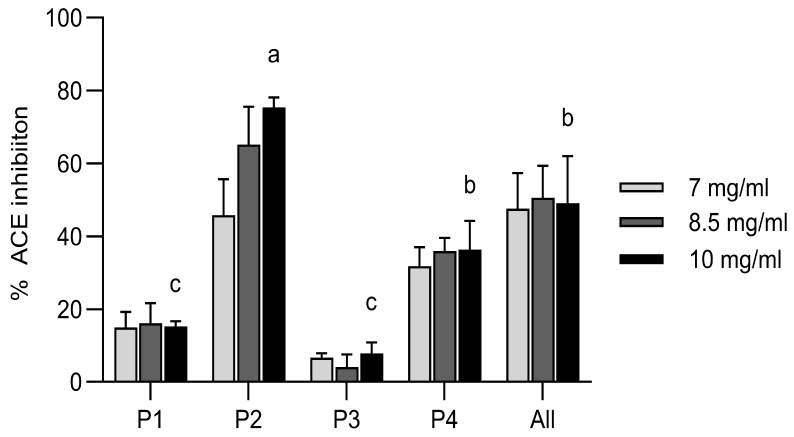
Angiotensin-I converting enzyme inhibitory activity *in vitro* of the custom synthesized sea cucumber peptides from *P. tremulus*, as determined using an enzymatic assay. Shown is the mean % of ACE I inhibition for three replicates at three tested concentrations (7, 8.5, and 10 mg/mL). Statistical differences were calculated only for results at 10 mg/mL by ANOVA, followed by the Holm–Šídák method. The same superscript letter means that the difference between the samples is not significant, while bars with different superscript letters are significantly different from each other (*p* < 0.05). A positive linear regression line over the tested concentrations could only be obtained for P2 and P4, allowing the calculation of IC50 values.

*In silico* modelling has also predicted the anti-hypertensive activity of the synthesized peptides, which was experimentally assessed as their ability to inhibit the ACE I-catalyzed cleavage of the substrate FA-PGG. Only for P2 and P4 could a positive linear regression line be obtained based on their ACE I inhibition (%) at the assessed concentrations. Their IC 50 values were calculated as 7.1 ± 0.8 mg/mL (4.5 ± 0.5 mM) and 26.3 ± 20.0 mg/mL (8.57 ± 6.5 mM), respectively (Figure 5). P2 showed by far the strongest ACE I inhibition with 75% at 10 mg/mL (Figure 5). This ACE I inhibition is slightly weaker than that reported for a *C. frondosa* body wall hydrolysate with an IC50 of 1.66 mg/mL [18] or a *Holothuria atra* hydrolysate with an IC50 of 0.32 mg/mL [38]. An isolated peptide from hydrolyzed *A. japonicus* gonads has shown ACE I inhibition with an IC50 of 260.2 ± 3.7 μM [39] and an isolated angiotensin-I converting enzyme inhibitory peptide from *Acaudina molpadioidea* gelatin had an IC50 of 14.2 µg/mL [40]. Although there was no synergistic effect of the combination of all the peptides seen in the bioactivity assays, their combination is still interesting in terms of their complementary behavior. Likewise, the peptide P2 with the overall lowest antioxidative capacity in the tested systems had the highest anti-hypertensive activity. It has still to be considered that all of these *in vitro* assessments have a rather low correlation to the assessment in the actual cell cultures *in vitro* or *in vivo* [41]. Also, a protective matrix might be needed if these effects are intended to be preserved in food applications. On the other hand, it has been shown that the antioxidative capacities might increase during digestion and fermentation [42,43]. Thus, it could be interesting to further assess the bioactivities from the peptide fragments after simulated human digestion, which might also be released in such conditions from the derived gel structures of a food product [15].

#### 2.4.3. Oxidative Stress Protection in Caco-2 Cell Culture Model

**Figure 6:**
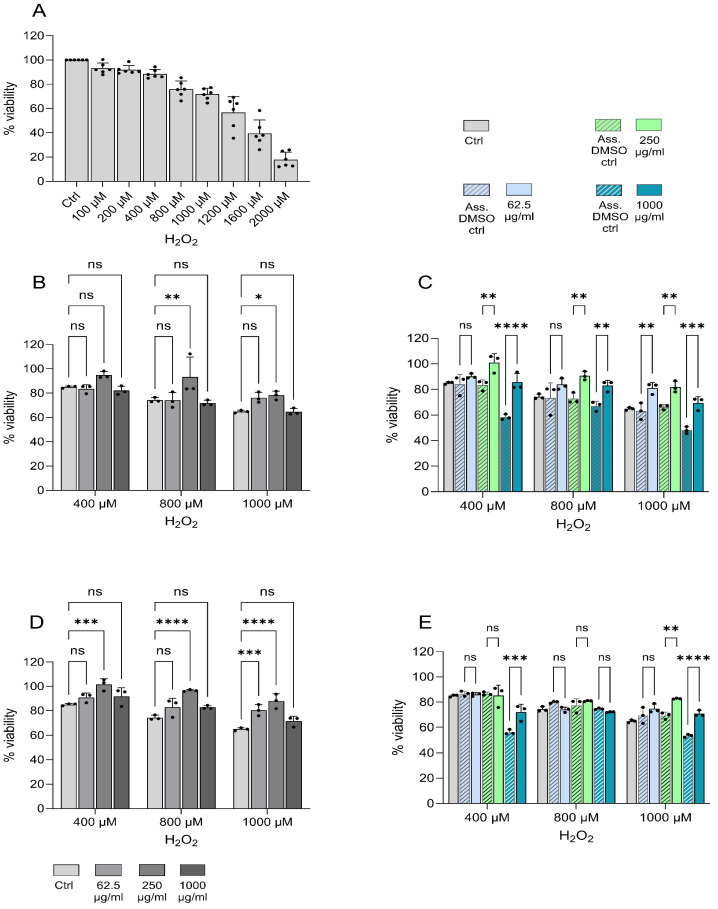
Oxidative stress protection capacity of the synthesized sea cucumber peptides from *P. tremulus*, as determined in a cell viability assay *in vitro*. Caco-2 cells were pre-protected with peptides at different concentrations (62.5, 250, and 1000 µg/mL) prior to induced oxidative stress using increasing concentrations of H_2_O_2_. (**A**) H_2_O_2_ treatment alone; (**B**) P1 + H_2_O_2_; (**C**) P2 + H_2_O_2_; (**D**) P3 + H_2_O_2_; and (**E**) P4 + H_2_O_2_. Since P2 and P3 were dissolved in DMSO, an additional DMSO control was added for each concentration of these peptides. The results are expressed as % cell viability compared with untreated control cells ± standard deviation of at least three replicates and differences were tested using ANOVA. The symbols above the bars correspond to ns = non-significant, * = *p*< 0.05, ** = *p* < 0.01, *** = *p* < 0.001 and **** = *p* < 0.0001.

The capacity of the peptides from *P. tremulus* to protect from oxidative stress-induced cell death was evaluated in H_2_O_2_-stimulated Caco-2 cells. First, different concentrations of hydrogen peroxide (H_2_O_2_) (100 µM–2000 µM) were added to the cells for 20 h before the cell viability analysis to determine at which concentration the H_2_O_2_-induced cell death is evident. The optimal range was between 400 µM and 1000 µM (Figure 6A) and was used in the successive experiments. The cells were pre-stimulated with peptides for 24 h before treatment with increasing concentrations of H_2_O_2_ (400, 800, and 1000 µM) for 20 h, followed by cell viability assays. The changes in cell viability are presented in Figure 6. Peptides P1 and P4 were dissolved in water, and their ability to protect cells from oxidative stress-induced cell death was compared with those treated with H2O2 alone (Figure 6B,D). The peptides P1 and P4 were dissolved in DMSO because they were insoluble in the aqueous cell culture medium. To rule out the potential antioxidant effect of DMSO, comparable DMSO amounts for each peptide concentration were added as internal controls. The change in cell viability was compared with the corresponding DMSO control for each peptide concentration (Figure 6C,E). All four peptides protected cells from oxidative stress-induced death, demonstrating the significant oxidative stress protection capacity of all the tested peptides, despite not being chosen for this study for their bioactivities. This result aligns with other studies performed in Caco-2 cells demonstrating bioactive peptides with antioxidant activity and oxidative stress protection capacity [44].

## 3. Conclusions

This study has contributed novel data on the bioactivity and assembly of four synthetic peptides, derived from sea cucumber body wall sequences, in fibers, in hydrophilic systems with the presence of calcium ions. With the results, we have advanced further the knowledge on marine natural peptide-based hydrogels, which could have the potential for upscaling to food or pharmaceutical product development. As the initial steps of the hydrogel structural characterization, we have microscopically confirmed the formation of fibrillar/laminar structures by the mixture of four peptides from the sea cucumber *P. tremulus* body wall. This is the first report providing experimental data on these four *P. tremulus* peptides, which could be successfully synthesized, were stable for up to one year in frozen conditions and could self-form (separately or in a mixture of four) gel structures. No application of additional treatment or the incorporation of high concentrations of chemicals was proven necessary. We have established some critical gelation conditions, such as minimal peptide concentrations, the presence of ligands, and have experimentally confirmed some of the bioactivities of the peptides *in vitro*, on a human intestinal epithelium cell culture. More research is needed to define the exact calcium binding capacity of each peptide and the structures of the formed multimeric complexes, the necessary modifications to allow for controlled physico-mechanical properties (depending on the sought-after application), and the bioavailability of the different possible peptide complexes for food hydrocolloid formulations. The exact structural organization of the peptide complexes should be further determined using higher resolution analytical techniques like circular dichroism and Fourier-transform infrared spectroscopy. The potential de novo allergenicity of sea cucumber peptide-based hydrogels should also be studied in the future.

## 4. Materials and Methods

### 4.1. Peptide Synthesis

The four peptide sequences from the sea cucumber *P. tremulus* selected for custom synthesis are presented in Table 1. The peptide synthesis was carried out using ProteoGenix SAS, (Schiltigheim France), followed by acetate N-terminal blocking and chromatography purification (LC-MS purity chromatograms per peptide are included in the Supplementary Materials). Thus, 1 g of lyophilized powder at 80% or a higher purity (technical grade) in sterile plastic bottles was provided by ProteoGenix for further research. The peptides were stored at −20 °C, opened only prior to the dissolution preparation and used within 2 years from the date of provision. The accompanying manufacturer information advised resuspension with DMSO (Merck Sigma, Darmstadt, Germany) for peptides 1 and 4, due to poor water solubility.

### 4.2. Molecular Structure Modelling of the Peptide Complexes

Three different web-based software solutions were initially compared for the structure-folding prediction of the complexes to be formed in the solution among the studied peptide sequences, at a neutral pH. The peptide sequences were thus analyzed using the GalaxyHomomer/GalaxyHeteromer services of GalaxyWEB; https://galaxy.seoklab.org, (accessed at several dates in October 2022 and June 2023 [19]), the MOLSOFT LLC ICM-Browser v.3.9 (https://www.molsoft.com/icm_browser.html; accessed last in October 2023 [45]), and the Alpha Fold 3.0 (https://alphafoldserver.com, accessed on 27 October 2024 [17]). Structural visualization was first performed through Galaxy and MOLSOFT. The prediction quality assessment for further model selection was performed on the basis of the average cluster size, TongDock/DockQ-score, or lowest stabilization energy index (sOPEP), through long-range stabilized amino acid interactions [19,46]. Only the top-highest score or first clustering option from each prediction case was thus taken into further account. The predicted 2D and 3D structural models were compared among different software tools, on the basis of their scores. Individual peptide charges were calculated using Pep-Calc.com’s online peptide calculator [47] and compared with the previously obtained most probable mass spectrometry ion peaks [16]. The most probable structures from Galaxy/MOLSOFT (in the lack of stabilizing external compounds or metal ions) were summarized and compared with the prediction of the AlphaFold 3 modelling online—based service (https://alphafoldserver.com/welcome).

### 4.3. Establishing Minimal Gelation Conditions

For the gelation study, dissolution was tested in modified artificial sea water with pH 7.74 (composition as per [48], all salts at the “for analysis” grade, from Merck Sigma, Darmstadt, Germany), due to its content of Ca^2+^ ions (final concentration in the buffer was 11 mM; Ca^2+^ previously established as a key gelation factor [16]) and its closeness to the native physico-chemical environment of the sea cucumber body wall proteins. Also, due to the preference for aqueous buffer systems for bioactivity testing (Section 4.5) and comments from the custom peptide producer, the solubility of the peptides was assessed in a 75 mM sodium phosphate buffer, pH 7.4. All the buffers were autoclaved for 20 min at 120 °C, in a VAPOUR-LINE Lite autoclave (VWR Chemicals International, Leuven, Belgium), prior to use. A range of concentrations of the peptides between 1.5 and 30 mM was tested for establishing the minimal gelation concentrations, for each of the peptides, and in a combination of all four (1:1:1:1 ratio). DMSO (Merck Sigma, D8418, Darmstadt, Germany) was used for better solubilization and effect upon gelation. All the experiments were carried out at room temperature (21 °C), in three repetitions. Optical solution (sol)-gel transitions were observed and documented. The resulting gels were further freeze-dried in the Labconco FreeZone 12 Liter equipment (Fisher Scientific, Loughborough, UK) and submitted to an SEM analysis as per the protocols detailed in the next section of this methodology.

### 4.4. Structural Characterization of Sea Cucumber Peptide Gels

#### 4.4.1. Scanning Electron Microscopy Analyses

The initial peptide fraction from *P. tremulus* hydrolysate after FPLC purification [20] was freeze-dried and used as control for the cryo-scanning electron microscopy (cryo-SEM) analysis on the FEI Quanta FEG 250 equipment (FEI Europe, Eindhoven, The Netherlands) at the Laboratory of Advanced Microscopy, University of Zaragoza, Spain. The mixture of the four custom synthesized peptides, selected for this study, at a ratio of 1:1, was prepared at minimal gelation conditions (15 mM final concentration, in the presence of 11 mM Ca^2+^ ions) and then lyophilized. This mixed peptide fraction was analyzed using the same SEM equipment, after palladium coating. The Image J 1.54d software [49] was used in parallel with the corresponding per equipment SEM SmartSCAN analysis software, for the measurement of the particle sizes on all the micrographs.

The solutions of the individual peptides (at a 15 mM final concentration, in a Ca^2+^ containing buffer, with the addition of DMSO for improved solubility) were prepared and then freeze-dried. An SEM analysis of these samples was performed at the NTNU NanoFab facility, Trondheim, Norway, using the FEI Apreo Field Emission equipment (FEI Europe, Eindhoven, The Netherlands), after coating with gold. Image J 1.54d was used in parallel with the SEM equipment software’s measurements, for the determination of the particle sizes on all the micrographs.

#### 4.4.2. Ultraviolet–Visible (UV) Absorption Spectrum of the Gel

The four sea cucumber peptides, separately and as a mixture of all four in a 1:1:1:1 ratio, were suspended in artificial sea water (VWR, pH 8) at a 5 mg/mL final concentration. The UV absorption spectroscopy of the samples was scanned using an ultraviolet spectrophotometer (Shimadzu UV-Vis spectrophotometer UV Mini 1240, Shimadzu Corporation, Tokyo, Japan) in the wavelength range of 190–400 nm, with artificial marine water (VWR) used as a blank for background adjustment. The corresponding peaks were recorded in the equipment and used for further comparison and analysis.

### 4.5. Experimental Bioactivity Confirmation

#### 4.5.1. Antioxidant Capacity

- ORAC

The ORAC assay was performed according to the BioTek Application Note (BioTek Instruments, Agilent Technologies, Winooski, VT, USA) with modifications [50]. The peptides were initially dissolved at 10 mg/mL in three separate replicates. According to their solubility, peptides P1 and P3 were dissolved in 75 mM NaPB at pH 7.4, whereas P2 was dissolved in 100% DMSO and P4 in 30% DMSO in NaPB. For the combination of all the peptides, equal molar amounts were combined, resulting in a final concentration of 0.96 mM of each peptide. The peptides were further diluted to 1:100 in NaPB. Trolox^®^ (6-hydroxy-2, 5, 7, 8-tetramethylchroman-2-carboxylic acid) (Sigma Aldrich #238813) standards (100–6.25 µM), sodium fluorescein (SoF, Sigma Aldrich #F6377) working solution at 0.04 µM, and AAPH (2,2′-azobis (2-amidinopropane) dihydrochloride (Sigma Aldrich #440914) at 153 mM were prepared in NaPB. In a black microplate, 25 μL of standards, samples, or blanks, and 150 μL SoF working solution were incubated at 37 °C for 30 min in the dark, followed by the addition of a 25 μL AAPH solution and fluorescence measurements (485/20 ex., 528/20 em.) at 37 °C every 10 min for a total of 60 min in a Synergy HTX S1LFA plate reader (BioTek Instruments, Agilent Technologies, Winooski, VT, USA). The antioxidative activity was calculated as Trolox equivalents (TEs) according to a standard curve based on the area under the curve (AUC). The AUC values were calculated using the following formula and the AUC values from the blank measurements for each buffer were subtracted:

AUC = 0.5 + (R2/R1) + (R3/R1) + (R4/R1) + … + 0.5 × (Rn/R1)

where R1 is the first and Rn is the last fluorescence reading.

- ABTS

For the ABTS assay [51], the initial peptide solutions at 10 mg/mL as described for the ORAC assay were further diluted at 1:100 in 0.01 M phosphate buffered saline (PBS). 2,2′-azino-bis(3-ethylbenzothiazoline-6-sulfonic acid) diammonium salt (ABTS salt; Sigma Aldrich A1888) and potassium persulfate (K2S2O8; Sigma Aldrich 379824) were dissolved at 7 mM and 2.44 mM, respectively, in 50 mL 0.01 M PBS at room temperature in the dark for 16–24 h. The formed ABTS•+ radical was diluted with PBS to an absorbance of 0.7 ± 0.05 at 734 nm in a Synergy HTX S1LFA plate reader (BioTek Instruments, Agilent Technologies, Winooski, VT, USA). Trolox was prepared at 1 mM in PBS and diluted to 300, 250, 200, 150, 100, 50, and 20 µM for the standard curve. In total, 10 µL of all the samples and 190 µL of the ABTS•+ working solution were combined and the absorbance at 734 nm was measured after 30 min. The activity of the samples was calculated as TE based on the standard curve.

- DPPH

For the DPPH assay (according to [52]), all four peptides were dissolved in three independent replicates at 10 mg/mL in DMSO and combined at 0.96 mM of each peptide as described for the ORAC assay. 2,2-diphenyl-1-picrylhydrazyl (DPPH, Sigma Aldrich D9132) was dissolved at 0.15 mM in methanol. Trolox was chosen as the standard in similarity to the assessment of the peptide samples and prepared at 100 mM in 75 mM NaPB and further diluted to 300, 250, 200, 150, 100, 50, and 20 µM in dH_2_O for the standard curve. The peptide solutions were assessed as two-times dilutions in dH_2_O and 20 µL of all the samples were combined with 180 μL of the 0.15 mM DPPH working solution, incubated for 30 min in the dark at room temperature, and the absorbance was measured at 540 nm in a Synergy HTX S1LFA plate reader. The activity of the peptides was calculated as TE based on the standard curve.

#### 4.5.2. Angiotensin-I Converting Enzyme Inhibitory Activity Measurement

For the angiotensin-I converting enzyme (ACE I) inhibitory activity measurements, the peptides were dissolved as described for the ORAC assay [53]. As the highest possible concentration of the peptides without gel formation was 10 mg/mL and no activity could be detected at 5 mg/mL, the peptides were assessed at 7, 8.5, and 10 mg/mL in three independent replicates. The substrate N-[3-(2-furyl) acryloyl]-L-phenylalanyl-glycylglycine (FA-PGG; Sigma Aldrich F7131) was prepared at 0.88 mM in 50 mM Tris-Cl, pH 7.5, and 0.3 M NaCl. In 96-well plates, 10 µL of the samples and their respective buffers as controls were combined with 10 µL of ACE I (ACE; Sigma Aldrich A6778) at 0.25 U/mL and 150 µL of a preheated substrate solution. The absorbance was continuously measured every minute at 340 nm for 30 min in a Synergy HTX S1LFA plate reader. Linear regression curves were obtained for the decrease in absorbance over time for every sample and the % ACE I inhibition was calculated as:

% ACE I inhibition = (1 − (ΔA340 nm protein solution/ΔA340 nm negative control)) × 100

Captopril was used to verify the functionality of the assay, and a logarithmic inhibition curve was obtained using 340, 34, 17, and 8.5 µM captopril. IC 50 values were calculated for the peptides for which a positive linear regression line could be obtained based on their ACE inhibition (%) at the assessed concentrations.

#### 4.5.3. Oxidative Stress Protection Capacity Testing in a Caco-2 Cell Culture Model

Cells of the human colon adenocarcinoma cell line Caco-2 were obtained from the American Type Cell Culture Collection (ATCC, Manassas, VA, USA) and used for the testing of the protection capacity of the sea cucumber peptides against oxidative stress generated by hydrogen peroxide (H_2_O_2_) [42]. Caco-2 cells were maintained in a complete medium: Eagle’s medium (MEM) supplemented with 10% fetal bovine serum (Gibco, Life Technologies Ltd., Paisley, UK), 100 U penicillin + 100 µg streptomycin (Merck Sigma, Darmstadt, Germany), 1 mM sodium pyruvate (Merck Sigma, Darmstadt, Germany), and 1× non-essential amino acids (Gibco, Life Technologies Ltd., Paisley, UK), and cultured at 37 °C in 5% CO_2_. The cells were passaged every third day.

For the experiments, the concentration of Caco-2 cells was adjusted to 1 × 10^4^ cells/mL in a complete medium, seeded into 96-well plates with 100 µL per well, and cultured at a high humidity at 37 °C and 5% CO_2_. The following day, the cells were pre-protected with different concentrations of the sea cucumber peptides at concentrations of 62.5, 250, and 1000 µg/mL diluted in a complete medium for 24 h. Peptides 2 and 4 were dissolved in 100% DMSO prior to further dilution in a complete medium. Thereafter, different concentrations of H_2_O_2_ were tested (in the range of 100, 200, 400, 800, 1000, 1200, 1600, and 2000 µM) for 20 h, and the cell viability was determined at all these conditions, compared with non-treated Caco-2 cells. The cell viability was determined using the Invitrogen Presto Blue assay (Thermo Fischer Scientific, Eugene, OR, USA), as per the instructions of the manufacturer. The absorbance was measured at 570 and 600 nm.

### 4.6. Statistical Analysis

Statistical analyses were performed using the GraphPad Prism Software v.10.4.1. (GraphPad Software Inc., Boston, MA, USA), as well as using the Excel Stat data analysis tools of Microsoft Excel^®^ for Microsoft 365. The results are expressed as mean values ± standard deviation if nothing else is stated. Differences were tested using an ordinary one-way ANOVA, followed by a *t*-test and/or the Holm–Šídák method for multiple comparisons. Superscripts were generated using the GraphPad Prism Software v.10.4.1. Bars that share the same letter are not significantly different from each other, while bars with different superscript letters are significantly different from each other (*p* ≤ 0.05).

## Figures and Tables

**Table 1:** Summary of the initial information for the synthetic *P. tremulus* peptides, as provided by the custom synthesis provider. The calculated net charge and isoelectric point are performed considering an N-terminal acetylation and C-terminal—OH ion. The chromatography profiles of separate peptide purity are presented in Supplementary Figure S1.

Assigned Peptide Code	Sequence	MW [g/mol] and Reconstitution Advice	Purity (HPLC) [%]	Net Charge at pH 7	Isoelectric Point
Peptide 1 (P1)	Ac—EMLWLSDGSMGFAEDTDAAFLPGDTIFGRI	3305.64; ACN:H_2_O (1:3)	87.68	−5.99	2.60
Peptide 2 (P2)	Ac—RAGQPITAFLVRD	1485.68; ACN:H_2_O (1:4)	91.20	0.00	8.01
Peptide 3 (P3)	Ac—SRPSDPASAVAGEDYTGISRN	2192.25; ACN:H_2_O (1:5)	85.74	−2.00	3.64
Peptide 4 (P4)	Ac—QNGEYGCVADTPNLLYAFKILDYRQ	2934.23; ACN:H_2_O (1:3)	88.81	−2.04	3.64
Calculated net charge of the peptide mixture at pH 7:				−10.03	3.88

**Table 2:** Studied gelation conditions of the four peptides from *P. tremulus*. All the experiments were performed at room temperature (21 °C).

Peptide	Buffer	Solubility/ Cross Linking/Additive	Mixing/Vortexing (1 min) *	Peptide Concentration [mM]
1	Ca^2+^ containing (artificial sea water), pH 7.7	DMSO	+	1; 15; 30
2	Ca^2+^ containing (artificial sea water), pH 7.7	DMSO	++	1; 15; 30
3	Ca^2+^ containing (artificial sea water), pH 7.7	------	+	1; 15; 30
4	Ca^2+^ containing (artificial sea water), pH 7.7	DMSO	+	1; 15; 30
1 + 2 + 3 + 4 (1:1 ratio)	Ca^2+^ containing (artificial sea water), pH 7.7	--------	+	1; 15; 30
1	75 mM sodium phosphate buffer, pH 7.4	------	+	1.5; 7.5; 15
2	75 mM sodium phosphate buffer, pH 7.4	DMSO	++	1.5; 7.5; 15
3	75 mM sodium phosphate buffer, pH 7.4	------	+	1.5; 7.5; 15
4	75 mM sodium phosphate buffer, pH 7.4	------	++	1.5; 7.5; 15
1 + 2 + 3 + 4 (1:1:1:1)	75 mM sodium phosphate buffer, pH 7.4	DMSO	+	1.5; 15; 30

* two + signs represent two times vortexing for 1 min.

## Data Availability

The original contributions presented in this study are included in the article/Supplementary Material. Further inquiries can bef directed to the corresponding author.

## Supplementary Materials

The following supporting information can be downloaded at: https://www.mdpi.com/article/10.3390/gels11040280/s1, Figure S1: Chromatography profiles after HPLC reverse-phase purification of each of the four synthetic peptides, as provided by the custom synthesis provider PROTEOGENIX, Schiltigheim, France. (A) peptide 1; (B) peptide 2; (C) peptide 3; (D) peptide 4; Table S1: Summary of the size measurements performed using SEM microscopy and Image J 1.54d on at least five photos per sample, with at least five measurements per photo for both parameters’ length and cross section; Figure S2: The gels formed by the separate and the combinations of the four peptides at 15 mM concentration and in the presence of Ca^2+^ ions, at room temperature; Figure S3: Additional SEM images from the analyzed peptide gels; Figure S4: The UV-Vis spectra of the sea cucumber peptides in artificial sea water, at room temperature, measured in the wavelength range of 190–400 nm.

## Abbreviations

2,2′-azino-bis(3-ethylbenzothiazoline-6-sulfonic acid (ABTS); 2,2′-azobis(2-amidinopropane) dihydrochloride (AAPH); angiotensin-I converting enzyme (ACE I); American Type Cell Culture Collection (ATCC); area under the curve (AUC); calcium ions (Ca2+); carbon dioxide (CO2); Creative Commons Attribution (CC BY); Elix-grade distilled water (dH2O); dimethylformamide (DMF); dimethyl sulfoxide (DMSO); 2,2-diphenyl-1-picrylhydrazyl (DPPH); extracellular matrix (ECM); hydrogen peroxide (H2O2); glycosaminoglycan (GAG); decellularized extracellular matrix (dECM), half-maximal inhibitory concentration (IC50); interface predicted template modeling (ipTM); mutable collagenous tissue (MCT); matrikine (MK); sodium phosphate buffer (NaPB); novel stiffening factor (NSF); oxygen radical absorbance capacity (ORAC); P1, P2, P3, P4, P1–4—four different sea cucumber peptides used in this study and their mixture in 1:1 ratio; phosphate buffered saline (PBS); Worldwide Protein Data Bank (wwPDB); Research Collaboratory for Structural Bioinformatics Protein Data Bank (RCSB PDB); lowest stabilization energy index (sOPEP); scanning electron microscopy (SEM); transmission electron microscopy (TEM); template-based docking (TBD); Trolox^®^ (6-hydroxy-2, 5, 7, 8-tetramethylchroman-2-carboxylic acid); Trolox equivalent (TE).
