# Supplementary material for: Microstructure of Sea Cucumber Parastichopus tremulus Peptide Hydrogels and Bioactivity in Caco-2 Cell Culture Model
Source: Gels. 2025 Apr 8;11(4):280. doi: 10.3390/gels11040280 (PMC12026874; doi:10.3390/gels11040280)
Supplement: Supplementary file 1 [file gels-11-00280-s001.zip › gels-3485272-supplementary.pdf]

gels

Article

Microstructure of Sea Cucumber Parastichopus Tremulus Peptide Hydrogels and Bioactivity in Caco-2 Cell Culture Model

Miroslava Atanassova [1,*], Jennifer Mildenberger [1], Marianne Doré Hansen [1] and Tarmo Tamm [2]

[1] Møreforsking AS, NMK, Borgundvegen 340, 6009 Ålesund, Norway
[2] University of Tartu, Institute of Technology, Nooruse 1, Tartu, Estonia
* Correspondence: miroslava.atanassova@moreforsking.no

Keywords: sea cucumbers; de novo peptides; hydrogel formation; microstructure; electron microscopy; antioxidant activity; angiotensin-I converting enzyme-inhibitory activity; Caco-2; protection against oxidation in cell culture

The authors declare no conflict of interest

Data Availability Statement: Data will be provided upon requirement.

Supplementary Tables

Table S1. Summary of the size measurements done by SEM microscopy software and Image J 1.54d on at least 5 photos per sample, with at least 5 measurements per photo for both parameters' length and cross section. A) Mean values and standard deviation for each sample and parameter; B) Individual comparisons of one tail p values after two sample t-test assuming unequal variances (statistics done by Excel Stat).

Sample	Mean Length Size ± stdev [μm]	Mean Cross Section Size ± stdev [μm]
**Mixed fraction after FPLC**	1.69 ± 1.0	0.80 ± 1.0
**Mixed fraction peptides 1-4**	26.99 ± 20.7	2.78 ± 2.3
**Peptide 1**	4.29 ± 5.6	2.23 ± 3.9
**Peptide 2**	11.24 ± 11.7	2.04 ± 1.9
**Peptide 3**	17.68 ± 14.1	3.33 ± 2.9
**Peptide 4**	12.41 ± 16.3	4.28 ± 7.6

A)

P (T<=t) *one-tail (length)	FPLC fraction	Mix 4 peptides	Peptide 1	Peptide 2	Peptide 3	Peptide 4
**FPLC fraction**	---------	1.023E-08	0,015	9.990E-06	7.720E-06	0.002
**Mix 4 peptides**	1.023E-08	---------	1.156E-07	0.000(1)	0.023	0.002
**Peptide 1**	0.015	1.156E-07	---------	0.002	9.197E-05	0.016
**Peptide 2**	9.990E-06	0.000(1)	0.002	---------	0.037	0.383
**Peptide 3**	7.720E-06	0.023	9.197E-05	0.037	---------	0.124
**Peptide 4**	0.002	0.002	0.016	0.383	0.124	----------

the cells with p≤ 0,05 are highlighted in light color

P* (T<=t) one-tail (cross section)	FPLC fraction	Mix 4 peptides	Peptide 1	Peptide 2	Peptide 3	Peptide 4
FPLC fraction	---------	1.562E-05	0,038	0,000(4)	9.357E-05	0,006
Mix 4 peptides	1.562E-05	---------	0.260	0.139	0.214	0.139
Peptide 1	0,038	0.260	---------	0.410	0.124	0.090
Peptide 2	0,000(4)	0.139	0.410	---------	0.026	0.052
Peptide 3	9.357E-05	0.214	0.124	0.026	---------	0.255

Peptide 4	0,006	0.139	0.090	0.052	0.255	---------

* the cells with p≤ 0,05 are highlighted in light color

B)

Supplementary Figures

Sample:	Peptide 1 Ac-EI-30
Lot. No.:	P211027-MX944280
Column:	Kromasil 100-5C18, 4.6*250mm, 5µm
Solvent A	A: 0.1% Trifluoroacetic Acid in 100% Acetonitrile
Solvent B	B: 0.1% Trifluoroacetic Acid in 100% Water

Gradient:		A	B
	0.0min	30%	70%
	25.0min	55%	45%
	25.1min	100%	0%
	30.0min	Stop	

Volume:	10µl
Wavelength:	220nm
Flow rate:	1.0ml/min

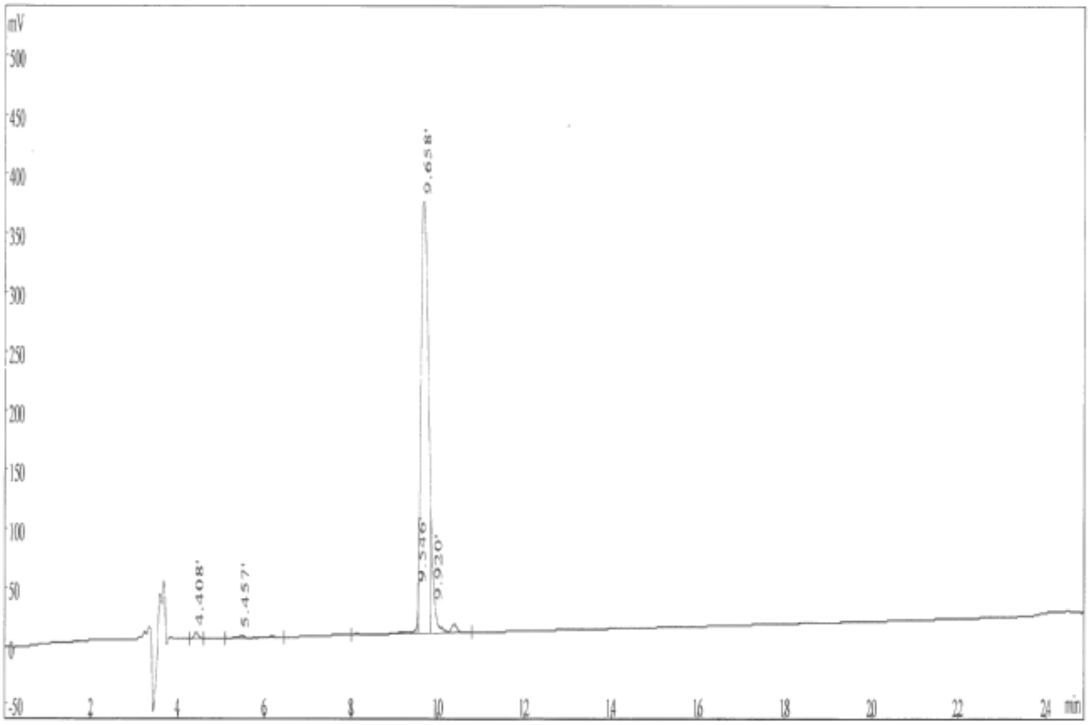

A)

Sample: Peptide 2 Sequence AC-RD-13

Lot. No.: P211027-MX944281

Column: Kromasil 100-5C18, 4.6*250mm, 5μm

Solvent A A: 0.1% Trifluoroacetic Acid in 100% Acetonitrile

Solvent B B: 0.1% Trifluoroacetic Acid in 100% Water

Gradient:

	A	B
0.0min	24%	76%
25.0min	49%	51%
25.1min	100%	0%
30.0min	Stop	

Volume: 10μl

Wavelength: 220nm

Flow rate: 1.0ml/min

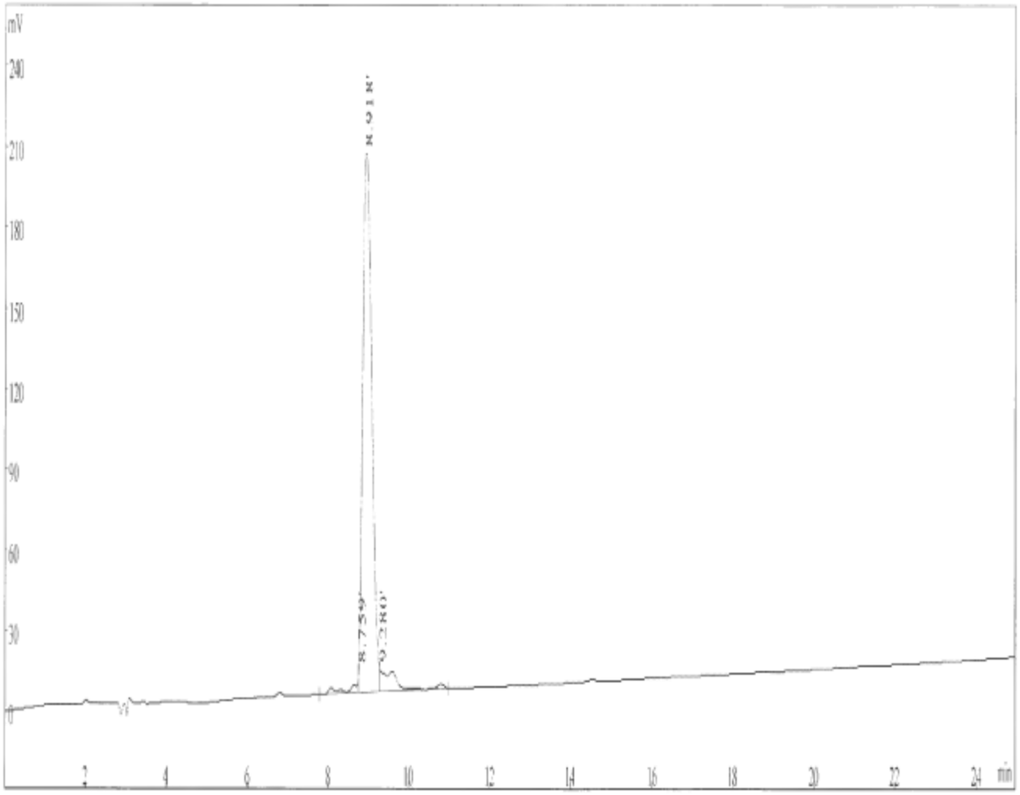

B)

Sample:	Peptide 3 Sequence AC-SN-21		
Lot. No.:	P211027-MX944282		
Column:	Kromasil 100-5C18, 4.6*250mm, 5μm		
Solvent A	A: 0.1% Trifluoroacetic Acid in 100% Acetonitrile		
Solvent B	B: 0.1% Trifluoroacetic Acid in 100% Water		
Gradient:		A	B
	0.0min	15%	85%
	25.0min	40%	60%
	25.1min	100%	0%
	30.0min	Stop	
Volume:	10μl		
Wavelength:	220nm		
Flow rate:	1.0ml/min		

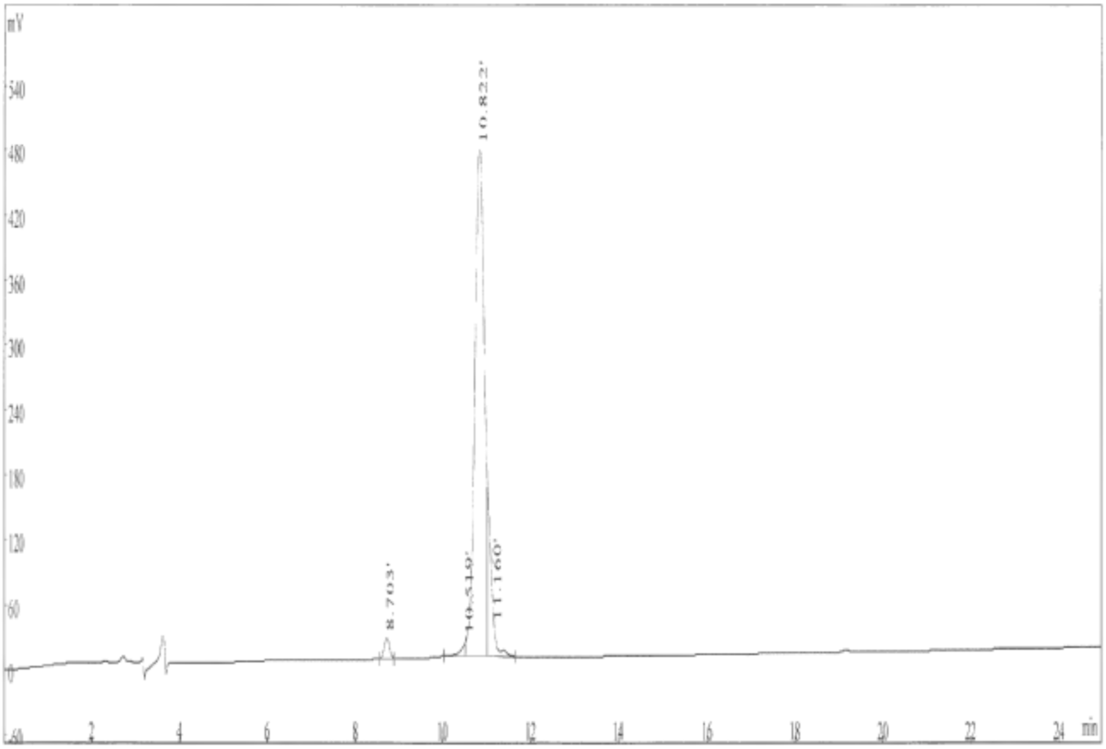

C)

Sample: Peptide 4 Sequence Ac-QQ-25
Lot. No.: P211027-MX944283
Column: Kromasil 100-5C18, 4.6*250mm, 5μm
Solvent A A: 0.1% Trifluoroacetic Acid in 100% Acetonitrile
Solvent B B: 0.1% Trifluoroacetic Acid in 100% Water
Gradient:

	A	B
0.0min	31%	69%
25.0min	56%	44%
25.1min	100%	0%
30.0min	Stop	

Volume: 10μl
Wavelength: 220nm
Flow rate: 1.0ml/min

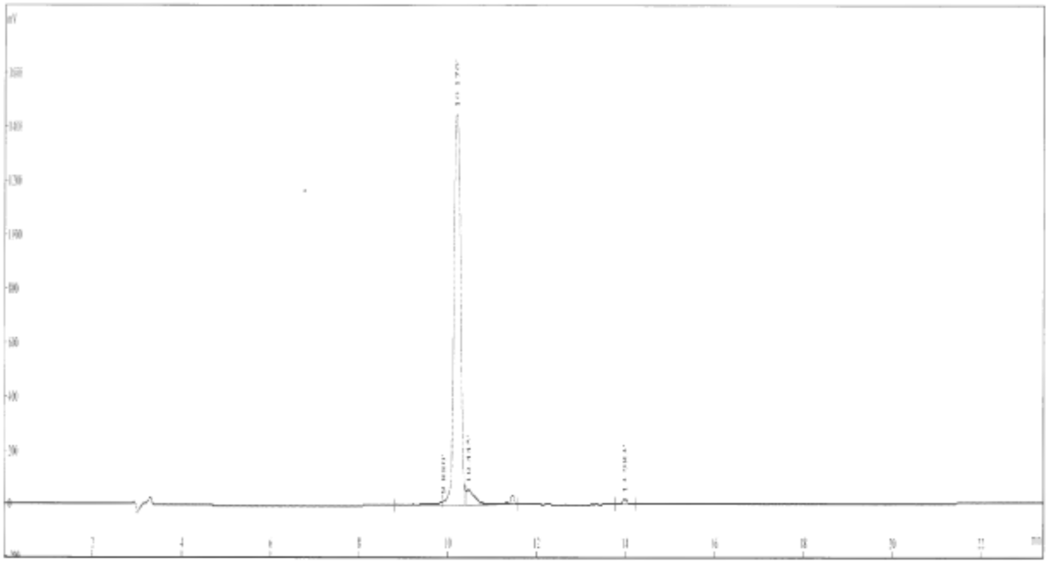

D)

Supplementary Figure S1. Chromatography profiles after HPLC reverse phase purification of each of the four synthetic peptides, as provided by the custom synthesis provider PROTEOGENIX, Schiltigheim, France. A) peptide 1; B) peptide 2; C) peptide 3; D) peptide 4.

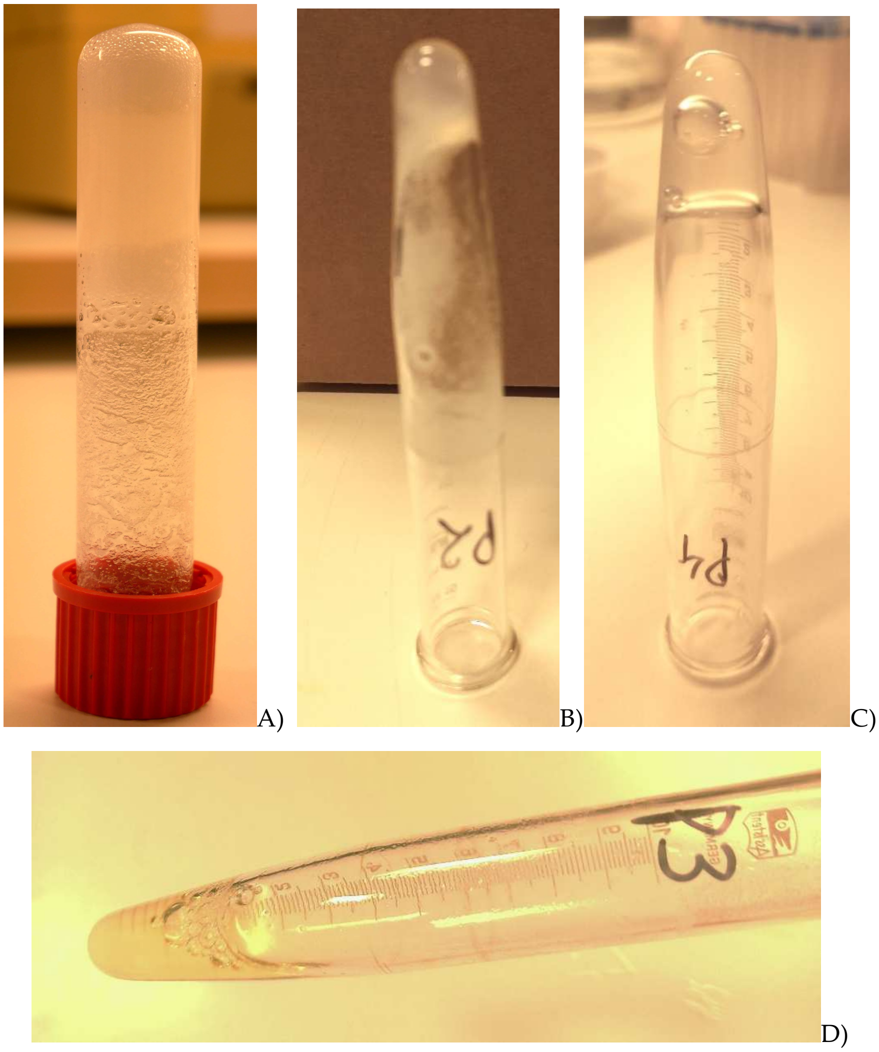

Supplementary Figure S2. The gels formed by the separate and the combination of the four peptides at 15mM concentration and in presence of Ca2+ ions, at room temperature. A) The mixed gel obtained after combining peptides P1 to P4 in gellation conditions; B) Solid gel obtained from P2 in gellation conditions; C) Solid gel obtained from P4 in gellation conditions; D) Liquid, highly viscous gel from P3 in gelation conditions, as example for the hydrogels obtained from P1 and P3 at the established gelation conditions.

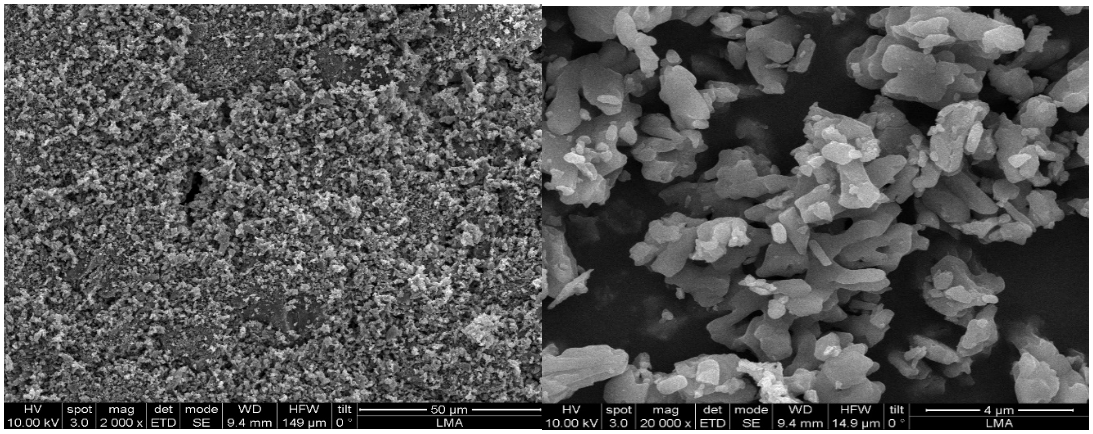
HV 10.00 kV spot 3.0 mag 2 000 x det ETD mode SE WD 9.4 mm HFW 149 µm tilt 0 °
50 µm
LMA
HV 10.00 kV spot 3.0 mag 20 000 x det ETD mode SE WD 9.4 mm HFW 14.9 µm tilt 0 °
4 µm
LMA

A)

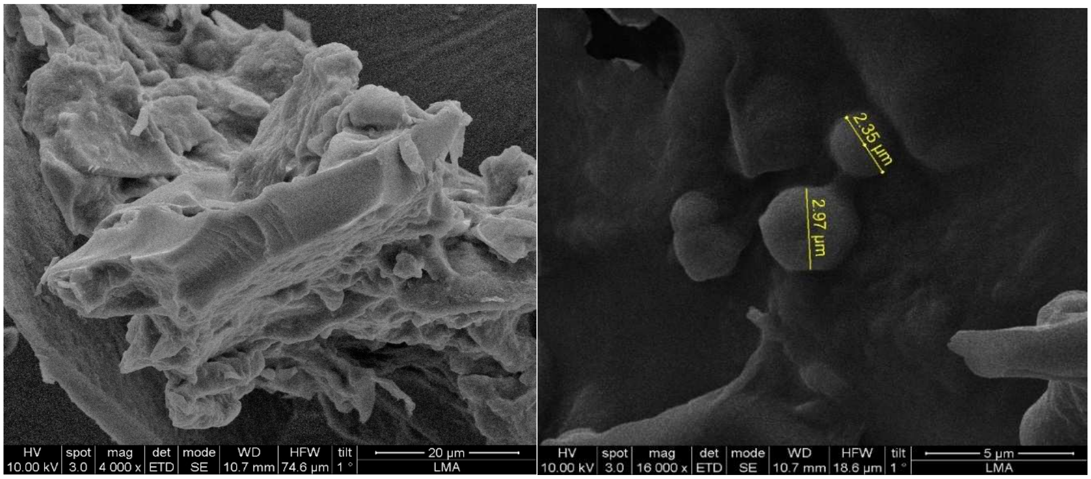
HV 10.00 kV spot 3.0 mag 4 000 x det ETD mode SE WD 10.7 mm HFW 74.6 µm tilt 1 °
20 µm
LMA
2.35 µm
2.97 µm
HV 10.00 kV spot 3.0 mag 16 000 x det ETD mode SE WD 10.7 mm HFW 18.6 µm tilt 1 °
5 µm
LMA

B)

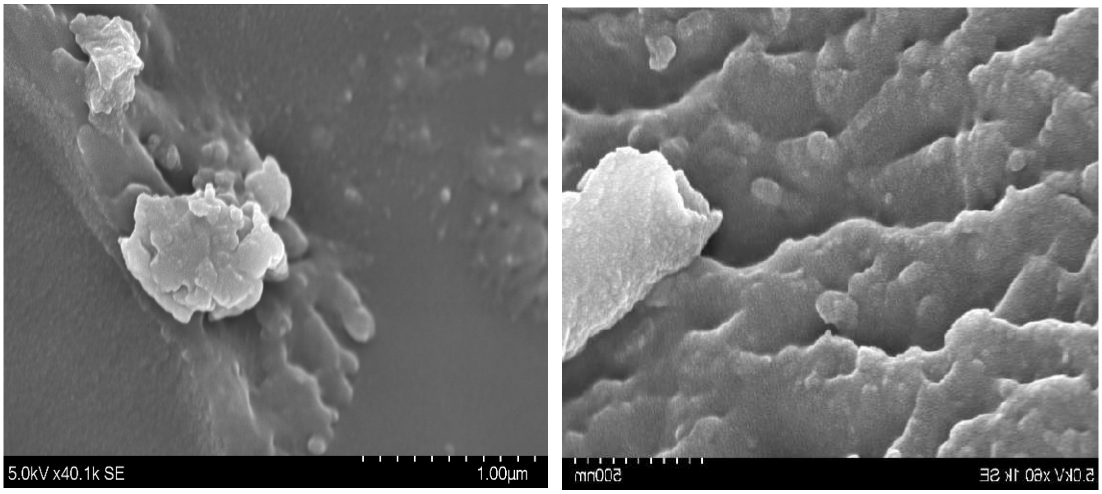
5.0kV x40.1k SE
1.00µm

C)

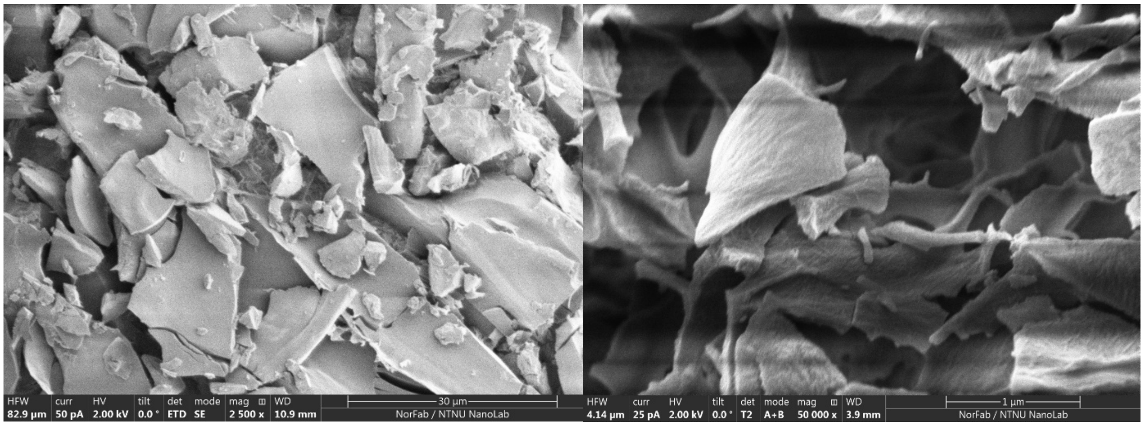

D)

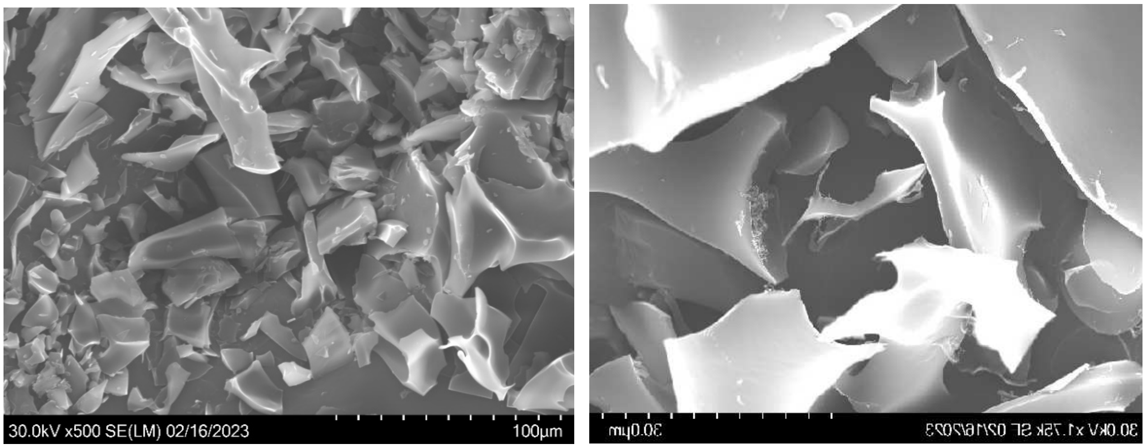

E)

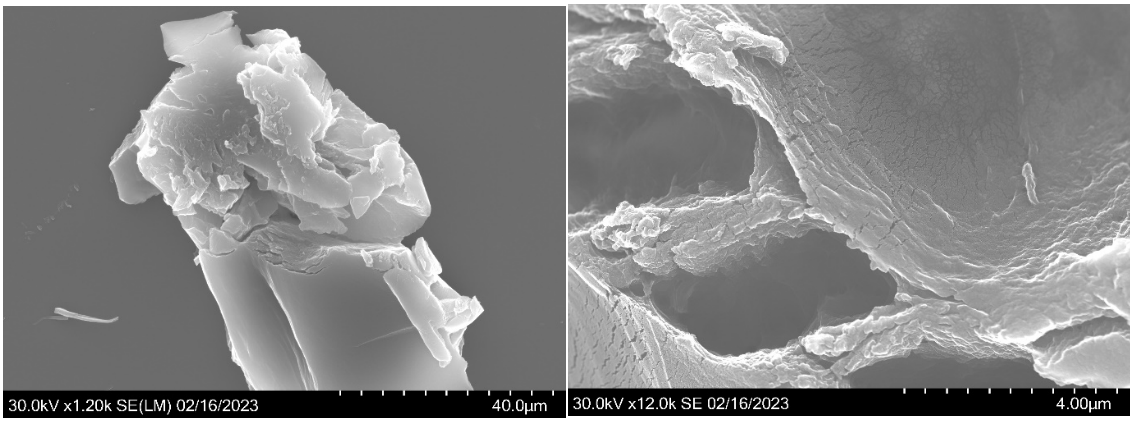

F)

Supplementary Figure S3. Additional fields of view to the SEM images from the analyzed lyophilized initial protein and peptide samples (at gelation concentrations for the latter), presented in Figures 2 and 3. A) FPLC protein fraction used for the definition of the peptide sequences of interest; B) Mixed fraction of the four peptides (P1-4); C) Peptide 1; D) Peptide 2; E) Peptide 3; F) Peptide 4.

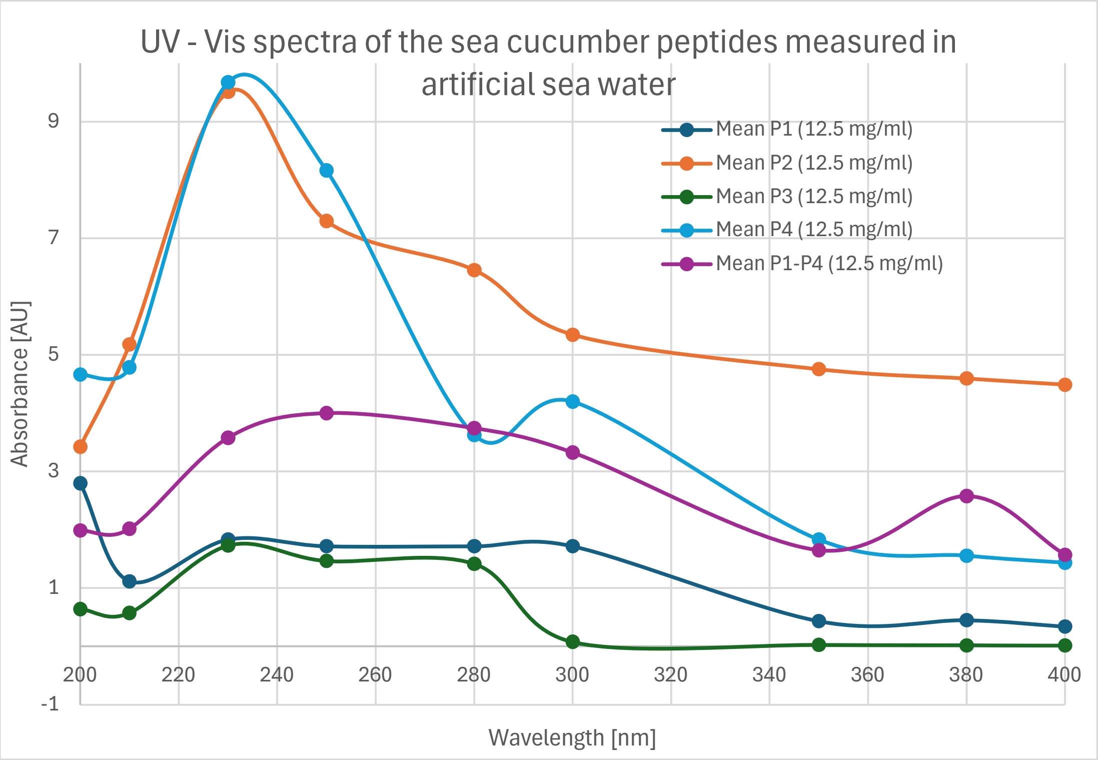

Supplementary Figure S4. Plot of the UV-Vis spectra of the studied four sea cucumber peptides in artificial sea water (presence of Ca2+ ions), at room temperature, measured in the wavelength range of 190-400 nm with a Shimadzu UV-Vis spectrophotometer UV Mini 1240. Absorbance is presented in arbitrary units [AU], since the values included in the Y axis are adjusted by calculation to account for equal sample concentrations for the different peptide dissolutions.
